# Present status of laboratory diagnosis of human taeniosis/cysticercosis in Europe

**DOI:** 10.1007/s10096-017-3029-1

**Published:** 2017-07-01

**Authors:** M. A. Gómez-Morales, T. Gárate, J. Blocher, B. Devleesschauwer, G. S. A. Smit, V. Schmidt, M. J. Perteguer, A. Ludovisi, E. Pozio, P. Dorny, S. Gabriël, A. S. Winkler

**Affiliations:** 1 Department of Infectious Diseases, Istituto Superiore di Sanità, viale Regina Elena 299, 00161 Rome, Italy; 2 Instituto de Salud Carlos III, Centro Nacional de Microbiología, Majadahonda, 28220 Madrid, Spain; 3 Institute of Acute Neurology, Academic Teaching Hospital Feldkirch, Carinagasse 47, 6800 Feldkirch, Austria; 4 Department of Public Health and Surveillance, Scientific Institute of Public Health (WIV-ISP), Rue Juliette Wytsmanstraat 14, 1050 Brussels, Belgium; 5 Department of Biomedical Sciences, Institute of Tropical Medicine, Nationalestraat 155, 2000 Antwerp, Belgium; 6 Faculty of Veterinary Medicine, Department of Virology, Parasitology and Immunology, Ghent University, Merelbeke, Belgium; 7 Institute of Health and Society (IRSS), Université Catholique de Louvain, Brussels, Belgium; 8 Department of Neurology, Klinikum rechts der Isar, Technical University Munich, Ismaninger Straße 22, 81675 Munich, Germany; 9 Centre for Global Health, Institute of Health and Society, University of Oslo, Kirkeveien 166, 0450 Oslo, Norway; 10 Faculty of Veterinary Medicine, Department of Veterinary Public Health and Food Safety, Ghent University, Ghent, Belgium

**Keywords:** Cysticercosis, Neurocysticercosis, *Taenia solium*, *Taenia saginata*, Taeniosis, Laboratory diagnosis

## Abstract

Human cysticercosis (CC) is a parasitic zoonosis caused by the larval stage (cyst) of the *Taenia solium*. Cysts can establish in the human central nervous system (neurocysticercosis, NCC) and other organs and tissues; they also develop in pigs, the natural intermediate host. Human taeniosis may be caused by *T. solium*, *Taenia saginata* and *Taenia asiatica* tapeworms*;* these infections are usually asymptomatic, but show a significant relevance as they perpetuate the parasites’ life cycle, and, in the case of *T. solium*, they are the origin of (N)CC. In European Union (EU) member states and associated countries, the occurrence of autochthonous *T. solium* cases is debated, and imported cases have significantly increased lately; the status of *T. asiatica* has been never reported, whereas *T. saginata* is prevalent and causes an economic impact due to condemned carcasses. Based on their effects on the EU society, the specific diagnosis of these pathologies is relevant for their prevention and control. The aims of this study were to know the diagnostic tests used in European laboratories for human taeniosis/cysticercosis by means of a questionnaire, to determine potential gaps in their detection, and to obtain preliminary data on the number of diagnosed taeniosis/CC cases.

**Electronic supplementary material:**

The online version of this article (doi:10.1007/s10096-017-3029-1) contains supplementary material, which is available to authorized users.

## Introduction

Human cysticercosis (CC) is a zoonotic parasitic infection caused by the larval stage (metacestode, cysticercus) of the pork tapeworm *Taenia solium*, formerly named *Cysticercus cellulosae*. These cysticerci establish in the human central nervous system (neurocysticercosis, NCC), eye, muscle, and, in rare cases, other tissues, and are a major cause of epilepsy in endemic low-income countries [1]. NCC is considered to be the most common helminth infection of the human nervous system [1, 2]. Humans acquire CC by ingesting *T. solium* eggs, released by themselves (auto- or self-infection) or by another tapeworm carrier [2], through fecal-oral contamination [3–6]. Humans are the unique *T. solium* definitive host (taeniosis), acquiring the infection by eating raw or undercooked pork harboring cysticerci; pigs are the natural intermediate host developing cysticerci by ingesting parasite eggs (porcine cysticercosis) on human feces. The maintenance of the life cycle requires a close association between humans and pigs [7]. Human taeniosis may also be caused by *Taenia saginata* and *Taenia asiatica* [8], of which the cysticerci only establish in cattle and pigs, respectively. So far, CC caused by *T. saginata* and *T. asiatica* has never been reported in humans.

Human CC occurs globally and continues to cause serious health problems [9]. The highest rates of *T. solium* CC are found in areas of Latin America, Asia, and sub-Saharan Africa with poor sanitation and free-ranging pigs that have access to human feces [10, 11]. In the European Union member states and associated countries (henceforth EU), *T. solium* was endemic in the past, although recent publications suggest that autochthonous cases may still be possible in some regions [12–14]. In recent years, imported CC cases have increased in parallel to the increased migration and travel [15]. Human taeniosis is not associated with major clinical symptoms, but has significant implications as it perpetuates the parasites’ life cycle, and, in the case of *T. solium*, causes a risk of NCC in the tapeworm carriers and people in their environment. *T. solium* infection is consistently classified as the most relevant food-borne parasite worldwide [16, 17]. *T. saginata* causes economic loss in the bovine meat sector due to condemned carcasses [18, 19].

Based on its rather rare occurrence in the EU, NCC is a challenge for care providers. NCC clinical manifestations are pleomorphic, varied and nonspecific, being related to individual differences in the number, size, location, stage of the parasite(s) and in the severity of the host’s immune response to the parasite. Although no pathognomonic clinical picture exists, in endemic regions new onset epileptic seizures and progressive crescendo headache are highly suggestive of NCC. In non-endemic regions, the diagnosis of NCC is primarily based on neuroimaging, and confirmed/aided by serology [20, 21], whereas the detection of taeniosis is most commonly made by stool microscopic examination *(Taenia* genus specific). Nevertheless, the early and species-specific identification of the taeniid and subsequent adapted management is crucial to avoid not only human-to-human transmission, but also human-to-pig/cattle transmission. New diagnostic tools [22], more specific and sensitive (immunological and molecular assays), have recently been developed for taeniosis and cysticercosis, however they are not yet commercially available/widely used.

Therefore, the knowledge of the in vitro diagnostic tools used in the EU for the detection of taeniosis/cysticercosis and their performances, as well as the identification/mapping of EU laboratories carrying out specific diagnosis of the disease, is of particular importance for the control, management and surveillance of these parasitic diseases.

The overall aim of the present study was to find out more about the diagnostic tests used in EU laboratories for human taeniosis/cysticercosis by means of a questionnaire in order to: (i) identify the assays offered for their examination, (ii) determine potential gaps in the techniques used by comparison with recently developed tools, and (iii) have some preliminary data on the number of taeniosis and CC cases diagnosed in the laboratories of different EU countries. In the present work, with the term taeniosis, we refer only to infections caused by cestodes of the genus *Taenia*.

## Materials and methods

### Participants

CYSTINET, the European Network on Taeniosis/Cysticercosis, consists of 27 EU countries, two EU Associated countries (Norway and Switzerland), one country (Serbia) that initiated the Stabilization and Association Process, one country (the former Yugoslav Republic of Macedonia [FYROM]) that is a candidate for accession to EU, six international partner countries and the World Health Organization (WHO) as specific organization (http://www.cost.eu), corresponds to the COST Action TD1302 (http://www.cystinet.org/). All CYSTINET members were invited via e-mail and orally at two CYSTINET meetings to fill in or forward the questionnaire link to microbiology laboratories within their specific countries.

### Data collection

A set of multiple-choice questions was composed by the CYSTINET members to collect the information for the present study. Apart from some general information regarding laboratory and contact details, all questions referred to the current activity of the laboratory in the field of *T. solium* and *T. saginata* cysticercosis/taeniosis diagnostics. The questionnaire was pre-tested by CYSTINET laboratory members and thereafter finalized (Supporting information, S1 File). Since the questionnaire was composed by multiple-choice questions, the laboratory had to select the answer and if one was non-applicable, the subsequent questions related to the former remained closed. The internet-based questionnaire software SoSci Survey [23] was used to gather the data. Every laboratory could access the questionnaire with a link on the website www.soscisurvey.de. Data was downloaded from the server and processed using SPSS (SPSS Inc., released 2009, PASW Statistics for Windows, version 18.0, Chicago).

There were no restrictions for laboratories to access the questionnaire. All questionnaires were examined, including those that were not completely filled in and those from laboratories that did not agree to display their contact details. For duplicate entries, the duplicate with the least information was discarded. All answers were included anonymously in the study. Table 1 summarizes the main questions answered by the participant laboratories.

**Table 1:** Questionnaire composed by a set of multiple-choice questions to collect information about the current activity of the laboratory in the field of *T. solium* and *T. saginata* cysticercosis/taeniosis diseases

Questions	Questions (cont.)
What kind of samples can you test for *T. solium* taeniosis?	Do you use immunodiagnostic methods to approach *T. solium* cysticercosis?
What kind of samples can you test for *T. saginata* taeniosis?	Do you perform a *T. solium* cysticercosis antibody test?
How do you handle *T. saginata* taeniosis suspected samples?	Which antibody detecting methods do you use to approach *T. solium* cysticercosis?
Do you perform a *T. solium* taeniosis test?	Do you perform a *T. solium* cysticercosis antigen test?
Do you perform a *T. saginata* taeniosis test?	What kind of antigen do you use in your in-house antibody detection test?
Do you use microscopic methods to approach *T. solium* taeniosis?	Do you observe any relevant cross reactions in your cysticercosis antibody tests?
Do you use molecular methods to approach *T. solium* taeniosis?	Which antigen detection methods do you use to approach *T. solium* cysticercosis?
Do you use immunodiagnostic methods to approach *T. solium* taeniosis?	What kind of antibodies do you use in your in-house test?
Do you observe any relevant cross reactions in your *T. solium* taeniosis tests?	Do you observe any relevant cross reaction in your cysticercosis antigen tests?
If you use any other test for *T. solium* taeniosis suspected samples, please specify	How do you evaluate sensitivity and specificity of your tests?
Do you use microscopic methods to approach *T. saginata* taeniosis?	Are there national ring-trials in your country to ascertain the quality of your *T. solium* and *T. saginata* tests?
Do you use molecular methods to approach *T. saginata* taeniosis?	Would you be interested in participating in a European ring trial for *T. solium* and *T. saginata* tests?
Do you use immunodiagnostic methods to approach *T. saginata* taeniosis?	Do you have diagnostic tests for other *Taenia* spp. available?
Do you observe any relevant cross reactions in *T. saginata* taeniosis tests?	Approximately how many *T. solium/saginata* taeniosis or cysticercosis positive samples did you have last year?
If you use any other test for *T. saginata* taeniosis suspected samples, please specify	Do you refer *T. solium/T.saginata* cysticercosis or taeniosis samples to other laboratories?
Which samples can you test for *T. solium* cysticercosis?	Where do you refer the *T. solium* (neuro)cysticercosis suspicious samples to?
Do you use molecular methods to approach *T. solium* cysticercosis?	Where do you refer the *T. solium*/saginata taeniosis suspicious samples to?

Based on the obtained information, an interactive map with the different institutions carrying out the diagnosis of the diseases in each country that agreed to display their information was made available through the COST action website (http://projects.cbra.be/cystinet/).

## Results

**Fig. 1:**
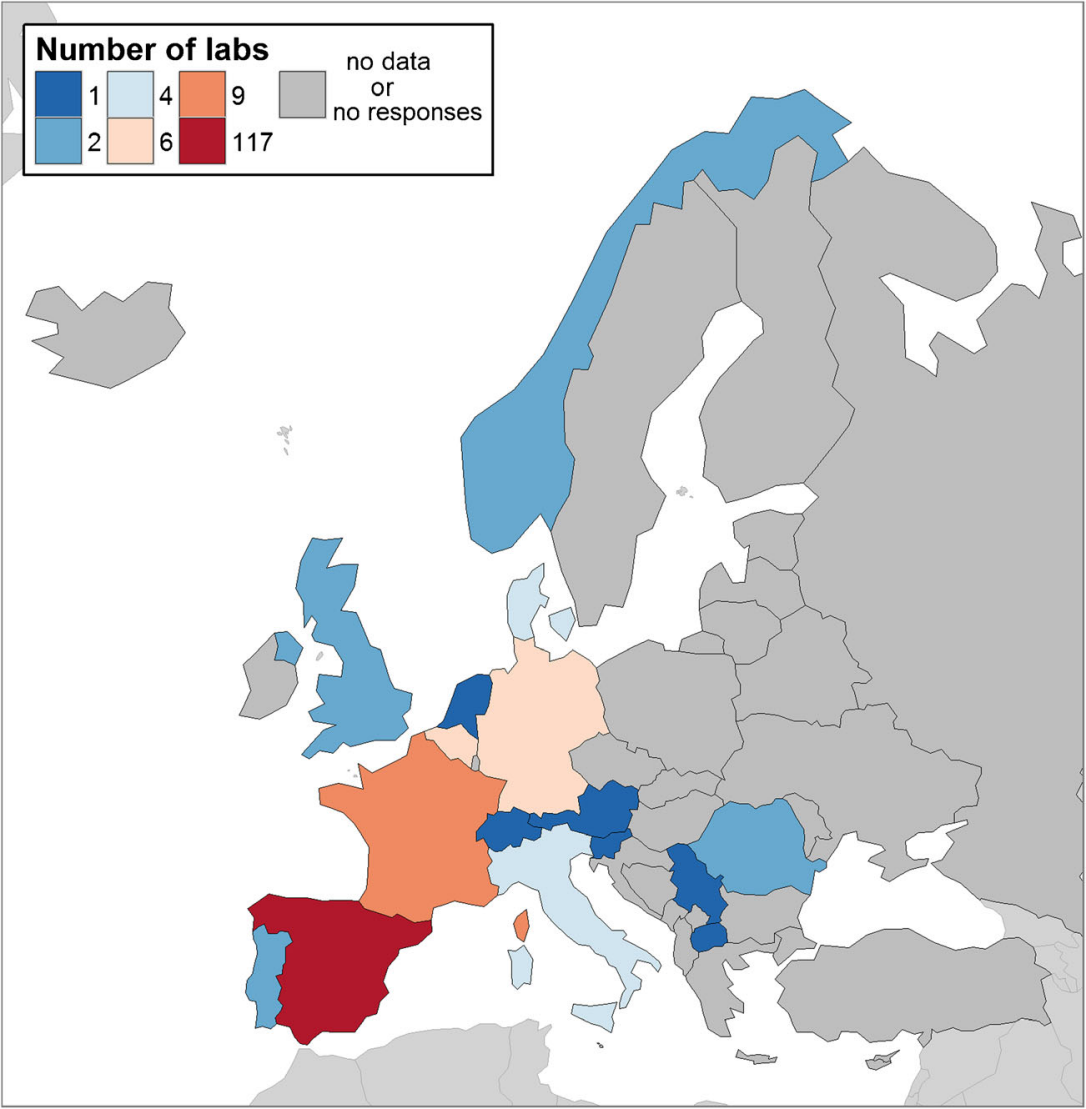
Number of respondent laboratories to the questionnaire survey among European Union member states and associated countries S1 File. Diagnostic questionnaire (PDF).

From August 2014 to February 2016, 160 laboratories filled in the questionnaire, but only 139 laboratories agreed to have their input published (Table 1). The respondents were from 16 European countries, with Spain being the country from which most responses (*n* = 117) were received (Fig. 1). Most of the laboratories were microbiology laboratories. Few of them were research laboratories, which also work as reference laboratories and/or public health institutions in their countries; therefore, they also have to support other laboratories for specific problems, such as taeniosis/cysticercosis diagnosis, *Trichinella* outbreaks, cystic echinococcosis diagnosis, etc.

### *T. solium* and *T. saginata* taeniosis diagnosis

Stool and proglottids were declared to be the samples mainly tested for the taeniosis diagnosis (Table 2A). Seventy-six laboratories (48%) stated that they handled *T. solium* and *T. saginata* taeniosis suspected samples in the same way and used the same tests, whereas eight (5%) laboratories declared they tested *T. saginata* and *T. solium* suspected samples, differently. Seventy-four laboratories (46%) did not provide any answer to this question.

**Table 2:** (A) Number of laboratories and samples matrices, and (B) Number of laboratories and specific tests used to establish a diagnosis of *T. solium* (neuro)cysticercosis and *T. solium/T. saginata* taeniosis

**A**					
Sample matrix tested	*T. solium* (neuro)cysticercosis		Taeniosis		
			*T. solium*	*T. saginata*	
Stool	0		81	10	
Proglottids	0		73	10	
Serum	38		28	1	
Cerebrospinal fluid	28		6	0	
Tissue	11		2	1	
**B**					
Tests			Pathology		
			*T. solium* (neuro)cysticercosis	Taeniosis	
				*T. solium*	*T. saginata*
Non specified				87	
Microscopy	Stool concentration			17	
	Stool concentration and morphological identification of the proglottids		0	29	5
	Fresh exam and morphological identification of the proglottids		0	41	2
	Other (Kato-Katz)		0	2	0
Immunodiagnosis	Antibody detection	ELISA	31	12	0
		WB	14	3	0
		IFA	2	0	0
	Antigen detection	In house	1	0	0
		Commercial	2	0	0
Molecular method		c-PCR	9	11	4
		RT-PCR	3	1	0
		c-PCR + Sequencing	0	2	0

*ELISA* enzyme-linked immunosorbent assay, *WB* western blotting, *IFA* immuno-fluorescence assay, *c-PCR* conventional polymerase chain reaction, *RT-PCR* real time polymerase chain reaction

Table 2B shows a summary of the tests used by the laboratories. Eighty-seven (54%) laboratories declared to test for taeniosis, whether caused by *T. solium* or *T. saginata*, but the method used was not specified. However, 78 (49%) laboratories stated the use of microscopy to diagnose taeniosis. The search for *T. solium* eggs in fecal samples by a microscope after stool concentration was carried out by 17 (22%) laboratories, one of which further used Ziehl-Neelsen staining [24]. Twenty-nine (37%) laboratories used stool concentration by formalin-ether or formalin-acetate as well as microscopic examination of the proglottids after ink staining. Forty-one (53%) laboratories reported to perform a microscopic examination of fresh fecal samples and a morphological identification of the proglottids; moreover, one laboratory used Carmine staining [25] and another declared to perform an ELISA as well. Two laboratories declared to use the Kato-Katz method; moreover, one of them reported to rely on histology and the other one on the cellophane-tape test [25]. For *T. saginata* taeniosis, five (50%) laboratories declared to perform stool concentration and two laboratories proglottid identification after ink staining [25].

Immunodiagnostic methods were declared to be used only for *T. solium* taeniosis in 14 (9%) laboratories, from which 11 declared to use ELISA on serum samples or whole blood, two western blot (WB) on serum samples and one ELISA plus WB on serum and cerebral spinal fluid (CSF). Seventy-one (44%) laboratories did not use any immunodiagnostic method to diagnose *T. solium* taeniosis and 75 (47%) did not provided any information.

Molecular methods were applied to both stool and proglottids by 15 laboratories. Nine (82%) laboratories employed conventional PCR (c-PCR) alone, c-PCR or real time PCR (RT-PCR) (one laboratory), and c-PCR and sequencing (two laboratories) for the *T. solium* taeniosis diagnosis. For *T. saginata* taeniosis, four laboratories declared using c-PCR.

### Other *Taenia* spp. diagnosis

Twenty-six out of 160 (16%) laboratories reported the availability of diagnostic tests for other *Taenia* spp. Fifteen out of 26 (58%) declared microscopy as the diagnostic tool employed. Two laboratories reported staining of the proglottids, and seven used PCR for all *Taenia* spp.

### *T. solium* (neuro)cysticercosis diagnosis

Serum and CSF were the preferred samples for *T. solium* (neuro)cysticercosis diagnosis (Table 2A), although tissue samples were also employed.

For *T. solium* (neuro)cysticercosis (Table 2B), 32 (20%) laboratories declared using immunodiagnostic methods based on antibody detection, and of those only three reported the use of antigen detection methods as well. Nine (28%) out of 32 laboratories used “in-house” ELISAs, from which six laboratories used crude extracts from whole cysticerci, three used cyst fluid, and one recombinant antigens as coating antigens. Commercial ELISA were from 11 companies and WB from five companies, kits were used by 28 and 14 laboratories, respectively. Indirect immuno-fluorescence assay (IFA) was employed by two laboratories, from which one declared using an “in house” IFA. An “in-house” antigen detection method for NCC, based on the monoclonal antibody, was used by one laboratory, and two laboratories stated using a commercial kit, which was specified only by one laboratory (Ag/ELISA ApDia).

Twelve (8%) laboratories declared using molecular methods for NCC; of them, nine (6%) used c-PCR and three (2%) RT-PCR in house tests. Molecular methods were used either on CSF (by two laboratories) or on cysts fluid (by one laboratory); the other nine laboratories did not specify the matrix used.

In addition, two (1.2%) out of 160 laboratories declared performing diagnosis on both bovine and porcine samples and 11 laboratories did not answer.

### Performance of taeniosis/cysticercosis tests and quality assurance

Fifteen (9%) laboratories underlined relevant cross reactions in NCC antibody tests used, but the names of the kits were not reported, whereas 15 (9%) did not observe cross reactions. Cross reactions were observed with *E. granulosus* infection (11 laboratories), *Entamoeba histolytica* (one laboratory), *Trichinella* spp. (one laboratory), *T. saginata* (one laboratory) and other non-specified helminths (three laboratories).

No cross reaction was observed in antigen detection methods for *T. solium* NCC. Forty (25%) laboratories reported no cross reactions in *T. solium* taeniosis tests, eight (5%) declared cross reactions with *E. granulosus* (two laboratories), other non-specified helminths (two laboratories) and *Entamoeba* spp. (one laboratory), or did not specify the cross reactive antigens. Eight laboratories reported no cross reaction in *T. saginata* tests, but all of them correctly underlined being unable to microscopically distinguish taeniid species by egg morphology.

Thirty (19%) laboratories evaluated the specificity and sensitivity of the tests relying on manufacturers’ information, 17 (11%) did an “in-house” evaluation and 10 (6%) performed different procedures, i.e. external quality control (five laboratories), inter-laboratory exchange (one laboratory), and literature-based evaluation (one laboratory). Fifty-six (35%) laboratories reported that they did not perform any control, and 17 (11%) laboratories ticked “unknown” with regard to tests sensitivity and specificity.

Concerning collaborative studies, 33 (21%) laboratories declared the existence of ring trials in their countries aimed at ascertaining the quality of the *T. solium* and *T. saginata* tests, although they did not confirm their participation. Eight (24%) laboratories reported the ring trials to be organized by a National Reference Laboratory and 16 by scientific societies. Ninety-four (59%) laboratories stated lack of awareness of the organization of ring trials in their countries and 34 did not answer. Forty-four (28%) laboratories declared not being interested in participating in a European ring trial for *T. solium* and *T. saginata* tests; whereas, 92 (58%) laboratories were interested in such a collaborative study, either for *T. solium* and *T. saginata* tests (80 laboratories) or for *T. solium* (12 laboratories) tests alone. Twenty-five (16%) laboratories did not provide any answer to this question.

Forty-eight (30%) laboratories reported refering all *T. solium/T. saginata* taeniosis or *T. solium* NCC suspicious samples to other laboratories. Sixty (37.5%) laboratories stated referring only some samples to other laboratories. Forty-five (28%) laboratories declared to never refer samples to other laboratories. Seven (4.3%) laboratories did not answer. Sixty-nine (43.1%) laboratories referred *T. solium* NCC suspicious samples to a reference laboratory and 19 (11.9%) to private laboratories. Seventy-five (46.9%) and 12 (7.5%) laboratories referred *T. solium/T. saginata* taeniosis suspicious samples to a reference laboratory, or to private laboratories, respectively.

The reported reference laboratories were: (i) the Institute of Tropical Medicine, Antwerp, in Belgium, (ii) the Statens Serum Institut, København, in Denmark, (iii) the Department of Infectious Diseases and Tropical Medicine, University Hospital Ludwig-Maximilians-Universität, Munich, and Bernhard-Nocht Institute for Tropical Medicine, Hamburg, in Germany, (iv) the Istituto Superiore di Sanità, Rome, in Italy, (v) the Mikrobiologisk Avdeling Haukeland Universitetssykehus, Bergen, in Norway, (vi) the Instituto de Salud Carlos III, Majadahonda; Hospital Miguel Servet, Zaragoza; Hospital Universitario Virgen de la Arrixaca, Murcia; Hospital Son Espases, Mallorca; Laboratorio de Referencia de Catalunya, El Prat de Llobregat; Hospital La Fe, and Hospital Clínico, Valencia; Hospital Virgen de la Victoria, Málaga; Hospital de Basurto, Bilbao, in Spain, (vii) the Clinical Hospital of Infectious Diseases, Cluj-Napoca, in Romania, and (viii) the Hospital for Tropical Diseases, London, in the United Kingdom.

### Positive samples of taeniosis and cysticercosis diagnosed during a one-year period

Twenty-four (15%) and 26 (16%) laboratories, tested NCC and taeniosis samples in the previous year, respectively (Table 3). Moreover, 54 (34%) laboratories declared to have had *T. saginata* taeniosis samples in the same time interval. Ninety (56%), 95 (59%) and 61 (38%) laboratories did not receive any sample for NCC, *T. solium* taeniosis, and *T. saginata* taeniosis, respectively, in the course of the previous year. The number of positive samples reported is shown in Table 3. Fifteen (9%), 13 (8%) and 9 (6%) laboratories declared being unaware of the number of NCC, *T. solium* taeniosis, and *T. saginata* taeniosis positive samples received.

**Table 3:** Number of positive *T. solium* (neuro)cysticercosis and *T. solium/T. saginata* taeniosis samples in the previous calendar year declared by the participating laboratories

Pathology	Number of positive cases in the last year	Number of laboratories	Country
*T. solium* (neuro)cysticercosis	Unknown	15	France, Norway, Romania, Spain
	No cases	90	Belgium, Denmark, France, Germany, Italy, FYROM, Norway, Portugal, Spain, UK
	1–5	18	Denmark, France, Germany, Spain, UK
	6–10	5	Germany, Spain, The Netherlands
	51–100	1	Spain
*T. solium* taeniosis	Unknown	13	France, Germany, Norway, Romania, Spain
	No cases	95	Belgium, Denmark, France, Germany, Italy, FYROM, Norway, Portugal, Spain, The Netherlands, UK
	1–5	23	Belgium, Germany, Serbia, Slovenia, Spain, Switzerland, UK
	6–10	2	Spain
	11–50	1	Spain
*T. saginata* taeniosis	Unknown	9	France, Norway, Rumania, Spain
	No cases	61	Belgium, Germany, FYROM, Italy, Portugal, Spain
	1–5	50	Belgium, Denmark, France, Germany, Norway, Spain, The Netherlands, UK
	6–10	4	Spain, UK

## Discussion

The diagnosis of *T. solium* (neuro)cysticercosis/*T. solium* taeniosis in immigrants and travelers from endemic regions, and sporadic autochthonous cases, continue to be a problem in the EU [12–14]. Moreover, *T. saginata* taeniosis persists, despite systematic meat inspection (64/433/EEC directive) [18], and the potential introduction of *T. asiatica* by immigrants and travelers from south-East Asia [26, 27] complicates the scenario. Since in vitro diagnostic tools are available for taeniosis and cysticercosis (Tables 4 and 5), the question arises whether laboratories from the EU are prepared to accurately diagnose human NCC and taeniid infections. Therefore, a questionnaire (Table 1) was prepared and distributed among EU laboratories to determine the present status of diagnostic tools used for the analysis of NCC, *T. solium* and *T. saginata* taeniosis.

**Table 4:** In vitro diagnostic tools for taeniosis

Tools	Samples	Comments
Microscopy	Feces (up to three specimens in different days), proglottids	• Gravid proglottids (count of uterine lateral branches) can allow species-specific identification of adult worms [25]. Taeniid eggs look identical irrespective of the species. • Other alternatives are needed to carry out the species-specific diagnosis of *T. solium* tapeworm carriers, when only taeniid eggs and/or poorly preserved proglottids are available.
Antibody detection	Serum	• Two recombinant antigens from *T. solium* adult worms (rTSES33 and rTSES38) [28] were cloned and expressed; rTSES33 [29] showed the best performance.
Antigen detection	Feces	• Coproantigen detection allows the detection before patency and evaluation of effectiveness of taenicidal treatment. • There are polyclonal antibody systems that do not exhibit taeniid species-specificity, like the enzyme linked immunosorbent assay (ELISA) for coproantigen [30] and/or the whole worm extract (WWE)-ELISA [31]. The most recent WWE- excretory secretory -ELISA [31] protocol showed a good sensitivity and an improved specificity for *T. solium* coproantigen-detection.
DNA detection	Feces, proglottids	• Excellent sensitivity and specificity on proglottids and stool samples from patent infections. The assay sensitivity will be related to the analytical sensitivity of the molecular target used [32–34], and its reliability will increase with the number of stool specimens analyzed (important to use an efficient DNA-extraction method for feces). It can also be applied to dubious proglottids. • Amplification protocols are the most used methods (PCRs), and are mainly based on the sequence of repetitive DNA, ribosomal DNA, mitochondrial DNA, and Antigen 2 (Ag2) gene [32–40]. Loop mediated isothermal amplification assays (LAMP) [41] could potentially overcome infrastructure challenges for the use of molecular techniques in endemic areas.

**Table 5:** In vitro diagnostic tools for cysticercosis

Tools	Samples	Comments
Microscopy	Biopsy	• Tissue examination by microscopy for the presence of suckers and hooks on the scolex [25].
Antibody detection	Serum, cerebrospinal fluid (CSF)	• Antibody detection in serum is the first option used in laboratories for neurocysticercosis (NCC) diagnosis. Antibodies indicate parasite-exposure and work better with active NCC in comparison to inactive NCC [42]. • Native antigens: the enzyme-linked immunoelectrotransfer blot (EITB) [43] assay with an affinity-purified lentil-lectin glycoprotein fraction (LLGP), used as antigens, is the gold-standard for NCC serodiagnosis. • Recombinant antigens and synthetic peptides: many genes encoding diagnostic antigens were described during recent decades [44–52], including the seven components of the LLGP fraction. They were cloned and expressed as recombinant proteins in both prokaryotic and eukaryotic systems, and evaluated using enzyme linked immunosorbent assay (ELISA), western blot (WB), immunochromatography, and others [53]. The most promising antigens are members of the 8 kDa diagnostic antigen family [44, 46, 50], used as fusion proteins/peptides, and recombinant proteins derived from the glycoproteins GPT24 and GP50 [47, 49, 54], employed independently or as “antigen cocktail”.
Antigen detection	CSF, serum	• The antigen-capture ELISAs based on the use of HP10 [55–57] or B158/B60 [58–61] monoclonal antibodies, prepared against *T. saginata* antigens, are the two diagnostic options employed in both detection and follow-up of NCC patients, mainly in patients with several active cysts and extraparenchymal locations, and in epidemiological studies in endemic regions.
DNA detection	CSF, biopsy	• The amplification protocols (polymerase chain reaction, PCR) are the same described for the taeniosis diagnosis (Table 4), but applied to CSF as clinical sample. So far, the amplification of pTsol9 repetitive sequence, in both conventional and real time PCR, and the seminested-HDP2-PCR, have already been used in NCC case identification [62–65].

The laboratories which filled in the questionnaire were randomly distributed in the EU (Fig. 1); indeed, most responders were from Spain (117 laboratories, 73%). Only one laboratory from Austria, Slovenia, Serbia, Switzerland and The Netherlands filled in the questionnaire. The high number of responding laboratories from Spain could be explained by an increasing interest on these pathologies due to the increase in imported cases in this country. Overall, the variation in responses could be due to (i) the different structure of health systems in the countries, more or less centralized according to the regions; (ii) lack of interest, due to the low number of cases of taeniosis/(neuro)cysticercosis diagnosed in some countries; (iii) problems with the adequacy of institutional servers, i.e. the questionnaire rejected by some of them; and (iv) no answer because of no proper distribution of the questionnaire.

As expected, stool and proglottids are the predominant samples tested for taeniosis, whereas serum is mainly tested for *T. solium* NCC. Several laboratories reported to employ tissue and CSF for NCC and few for *T. solium* taeniosis, whereas one laboratory stated testing biopsies and serum samples to diagnose *T. saginata* taeniosis (Table 2A). It must be stressed that biopsies, CSF and tissue samples are not adequate for taeniosis diagnosis [25]. These inadequate answers suggest that the question was not properly formulated and could have misled the participants, though the questionnaire was pre-tested; or it could be explained by a misunderstanding of these parasitic infections, with overlapping of the taeniid pathologies, or just by the lack of expertise in the diagnosis of intestinal parasites.

In general, EU laboratories seem better prepared for taeniosis diagnosis by microscopy than for (N)CC diagnosis by immunoassays [66]. This difference, observed also in the present study (Table 2B), could be explained by the low cost of basic coprological parasitological diagnostics that are routinely performed for the diagnosis of intestinal helminths, whereas “commercial” immunodetection test kits for (N)CC are expensive, have a limited shelf life, and are mainly available in laboratories, which receive a high number of requests in this specific diagnostic field.

Serological assays for *T. solium*, *T. saginata* and *T. asiatica* taeniosis diagnoses using recombinant antigens and immunoblots have been described [28, 67], but these assays are only used in research institutes and have not been commercialized yet.

### Taeniosis

Forty-eight percent of the respondent laboratories stated handling and processing *T. solium* and *T. saginata* taeniosis suspected samples in the same way with the same techniques, whereas 5% declared processing *T. saginata* and *T. solium* suspected samples differently. These data might indicate unawareness of the risk for the analysts in processing fecal samples potentially containing *T. solium* eggs. Containment Level 2 facilities, equipment, and operational practices are needed [68, 69].

With respect to the methodology employed for taeniosis diagnosis, as indicated above, microscopic methods were the option most frequently chosen as opposed to immunodiagnostic or DNA methods that were used in few laboratories only (Table 4). Copro-antigen detection was used mainly in research laboratories as this technique is not commercially available. Although copro-antigen detection is considered more sensitive than microscopy, its specificity is still a matter of debate [39, 70].

It is important to stress the relevance of molecular techniques for taeniid species identification [32, 34, 35]. In the case of feces containing eggs or proglottids, genomic amplification is the preferred diagnostic option for a differential identification and the best way to recognize *T. solium* carriers [39]. However, only few (7, 4.3%) laboratories reported to have molecular tools to distinguish the three *Taenia* spp. infecting humans [40, 71]. Identification at the species level is crucial because *T. solium* tapeworm carriers pose an immediate threat to themselves, their household members and close contacts. The diagnosis will determine the tapeworm-carrier management that should include treatment, parasite collection, and testing for NCC of both carrier and contacts. In the case of *T. saginata* tapeworm carriers, their management should include treatment, and safe disposal of the tapeworm collected to avoid environmental contamination leading to bovine infections. Considering the relevance of the species-specific identification, multiplexed amplification protocols [37] would be advisable, to avoid false negative results.

With regard to microscopy, only 21% of laboratories reported their participation in ring-trials, organized by National Reference Laboratories and Scientific Societies, to follow-up quality standards (http://www.instand-ev.de/en/news.html (INSTAND), Sociedad Española de Infección y Microbiología Clínica (SEIMC), Sociedad Valenciana de Microbiología Clínica (SVAMC), Norwegian Nasjonalt Folkehelseinstituttet (FHI)). These figures are low and they suggest the need for well-organized collaborative studies to evaluate the performance of taeniosis tests used by EU laboratories.

### Neurocysticercosis

Immunodiagnostic methods were employed by most of the laboratories rather than microscopy on biopsy samples or DNA detection methods for NCC (Table 5). Many laboratories used more than one test, frequently commercial kits, and few employed “in-house” assays [43, 72]. Regarding the specificity of these techniques, some laboratories highlighted relevant cross-reactions, with both protozoa and certain helminth species [73, 74], indicating no proper evaluation of the immunological tests used. Therefore, serological-ring trials with well-characterized clinical samples would be needed to determine the performances of the immunodiagnostic kits, to harmonize and standardize their use, and finally to know more about the real clinical significance of the immunodiagnostic tests employed for NCC [10, 54, 75]. Within immunodiagnostic methods, antigen-capture assays to diagnose NCC are a specific tool, mainly used on CSF. Among others, these assays allow identification and follow-up of complicated NCC infections [57, 76]. So far, the two options available were developed by European groups, the HP10 and the B158/B60 monoclonal antibody systems [77, 78], and are used for routine diagnosis and in epidemiological studies in endemic regions [60, 79].

Molecular techniques show a relevant sensitivity and excellent specificity in both taeniosis and NCC diagnosis, using different sample matrices such as CSF or tissue [40, 71]. However, the limited number of cases, lack of commercial kits and working infrastructure limit their use today.

In general, the number of positive NCC and *T. solium*/*T. saginata* taeniosis samples in the previous calendar year declared by the participating laboratories was low (Table 3). One laboratory from Spain reported the highest number of NCC and *T. solium* taeniosis cases (ranges from 51 to 100 and from 11 to 50, respectively). These data could indicate why the number of respondent laboratories was higher in Spain than in other European countries, because in this country the high number of Latin American immigrants could lead to an increased awareness and interest on the risk of imported *T. solium* human cases.

The implementation of routine ring trials, as some laboratories have already done, could be necessary to improve the standard quality level. In the case of NCC, a limited number (32, 20%) of laboratories use commercial kits and only few (9, 28%) of them use “in-house” tests that would require validation by well-organized collaborative studies.

Overall, there seem to be only a few (15, 9.3%) laboratories that have appropriate tools (all in vitro diagnostic approaches, including microscopy, and immunological and molecular assays) to identify *T. solium* taeniosis/NCC, which usually are located in European regions where *T. solium* taeniosis/NCC used to be endemic, where there is a strong travel/immigration pressure and/or where there are close relations with endemic areas by scientific networks. The results presented here are based on the replies of the participating laboratories, which were not evenly distributed over the involved countries, and as such the high number of laboratories responding from Spain has an influence on the results. The information obtained about the taeniosis/NCC tests used in the laboratories will be a valuable contribution for microbiology units to find support when they need it.

In addition, we suggest refreshing the knowledge on *T. solium* taeniosis/NCC infections as the prevalence of the disease seems rather low in Europe despite the fact that there is some evidence that NCC may actually be on the rise [80]. Some laboratories highlighted that they did not see cases anymore; however, we must be alert and ready for their diagnosis and surveillance. In the case of *T. saginata* infections, similar initiatives (e.g., knowledge refreshing on parasite transmission, risk factors, good practices, diagnostic tools) need to be applied, as *T. saginata* taeniosis persists in the EU countries despite an integrated approach among all stakeholders [18].

## Electronic supplementary material

ESM 1 (PDF 112 kb)
