# Supplementary material for: Present status of laboratory diagnosis of human taeniosis/cysticercosis in Europe
Source: Eur J Clin Microbiol Infect Dis. 2017 Jul 1;36(11):2029–40. doi: 10.1007/s10096-017-3029-1 (PMC5653711; doi:10.1007/s10096-017-3029-1)
Supplement: Supplementary file 1 — (PDF 112 kb) [file 10096_2017_3029_MOESM1_ESM.pdf]

Preview | [default] [barrier-free]

CYSTINET
EUROPEAN NETWORK ON TAENIOSIS/CYSTICERCOSIS

Print View from 08/08/2014, 13:25

Please note that filters and placeholders can not not work in the print view. Display of questions that are included via PHP code is limited.

Galley-proof Variable View [show PHP code]

Page 01

This Questionnaire was designed by CYSTINET members to find out more about diagnostic tests for T. solium/saginata taeniosis and cysticercosis.

Every laboratory, which performs tests for these diseases is invited to participate in the questionnaire.

The data collected will be analysed anonymously. If you agree, we will list your laboratory/insitution on the CYSTNET homepage. If not, we will not forward your contact details to anybody outside CYSTINET.

For further information visit our hompage: www.cystinet.org or contact us directly via e-mail: cystinet@gmail.com

You can pause and resume the questionnaire anytime by clicking on "Pause Questionnaire".

Page 02

Name of Department/Laboratory

Head of Deparmtent/Laboratory

Form filled in by (name):

1. Contact details:

Street_Number

Zip-code

City

Country

Additional information for address

e-mail

Phone

Fax

Website

2. Do you agree with the publication of your contact details on the CYSTINET homepage?

- Yes
- No

Page 03

We would like to make the questionnare as short as possible. Please answer all questions below and then we will select only the questions that are relevant for your laboratory/institute.

3. Do you perform a T. solium cysticercosis antibody test?

- Yes
- No

4. Do you perform a T. solium cysticercosis antigen test?

- Yes
- No

5. Do you perform a T. solium taeniosis test?

- Yes
- No

6. Do you perform a T. saginata taeniosis test?

- Yes
- No

7. Do you refer T. solium/T.saginata cysticercosis or taeniosis samples to other laboratories?

- ○ Yes
- ○ No

Page 04

question('SQ02')

8. Where do you refer the T. solium/saginata taeniosis suspicious samples to?

- ○ Send them to reference laboratory. Please indicate name and address: ____
- ○ Send them to private laboratory. Please indicate name and address: ____
- ○ We have no T. solium/saginata taeniosis suspected samples
- ○ Other: ____

question('SQ03')

9. Where do you refer the T. solium (neuro)cysticercosis suspicious samples to?

- ○ Send them to reference laboratory. Please indicate name and address: ____
- ○ Send them to private laboratory. Please indicate name and address: ____
- ○ We have no T. solium (neuro)cysticercosis suspicious samples
- ○ Other: ____

Page 05

question('SQ04')

10. What kind of samples can you test for T. solium taeniosis

- ☐ Stool
- ☐ Serum
- ☐ Proglotids
- ☐ Other: ____

question('SQ05')

11. Do you use microscopic methods to approach T. solium taeniosis?

- No
- Yes, please specify:

question('SQ06')

12. Do you use molecular methods to approach T. solium taeniosis?

- No
- Yes, please specify:

question('SQ07')

13. Do you use immunodiagnostic methods to approach T. solium taeniosis?

- No
- Yes, please specify:

question('SQ23')

14. Do you observe any relevant cross reactions in your T. solium taeniosis tests?

- No
- Yes, please provide further details:

question('SQ27')

15. If you use any other test for T. solium taeniosis suspected samples, please specify:

question('SQ26')

16. How do you handle T. saginata taeniosis suspected samples?

- ○ They are processed in the exact same way and we use the same techniques as for T. solium taeniosis suspected samples.
- ○ We process T. solium taeniosis suspected samples different than T. saginata suspected samples.

Page 06

question('SQ18')

17. What kind of samples can you test for T. saginata taeniosis?

- ☐ Stool
- ☐ Serum
- ☐ Proglotids
- ☐ Other: ______

question('SQ19')

18. Do you use microscopic methods to approach T. saginata taeniosis?

- ○ No
- ○ Yes, please specify: ______

question('SQ20')

19. Do you use molecular methods to approach T. saginata taeniosis?

- ○ No
- ○ Yes, please specify: ______

question('SQ21')

20. Do you use immunodiagnostic methods to approach T. saginata taeniosis?

- ○ No
- ○ Yes, please specify: ______

question('SQ24')

21. Do you observe any relevant cross reactions in T. saginata taeniosis tests?

- ○ No
- ○ Yes, please provide further details:

question('SQ28')

22. If you use any other test for T. saginata taeniosis suspected samples, please specify:

Page 07

question('SQ08')

23. Which samples can you test for T. solium cysticercosis?

- ☐ Serum
- ☐ CSF
- ☐ Tissue
- ☐ Other:

question('SQ09')

24. Do you use molecular methods to approach T. solium cysticercosis?

Tick all that apply.

- ☐ No
- ☐ PCR
- ☐ RT-PCR
- ☐ Other:

question('SQ10')

25. Do you use immunodiagnostic methods to approach T. solium cysticercosis?

- ○ No
- ○ Yes

question('SQ11')

26. Which antibody detecting methods do you use to approach T. solium cysticercosis?

Tick all that apply.

- ☐ No antibody detection.
- ☐ In-house ELISA
- ☐ Commercial ELISA. Please indicate name/company:
- ☐ In-house immunoblot
- ☐ Commercial immunoblot. Please indicate name/company:
- ☐ Other:

question('SQ12')

27. What kind of antigen do you use in your in-house antibody detection test?

Tick all that apply.

- ☐ No in-house test used
- ☐ Crude antigen from whole cysticerci
- ☐ Crude antigen from cyst fluid
- ☐ Glycoprotein fractions
- ☐ Recombinant antigens. Please specify:
- ☐ Other:

question('SQ22')

28. Do you observe any relevant cross reactions in your cysticercosis antibody tests?

- ○ No
- ○ Yes, please provide further details:

Page 08

question('SQ13')

29. Which antigen detection methods do you use to approach T. solium cysticercosis?

Tick all that apply.

- ☐ No
- ☐ In-house ELISA
- ☐ Commercial ELISA. Please indicate name/company:
- ☐ Other:

question('SQ14')

30. What kind of antibodies do you use in your in-house test?

Tick all that apply.

- ☐ No in-house test used
- ☐ Monoclonal antibodies. Please specify:
- ☐ Other:

question('SQ15')

31. Where are the monoclonal antibodies produced?

Tick all that apply.

- ☐ No monoclonal antibodies used.
- ☐ In-house
- ☐ By a commercial company. Please specify name of company:
- ☐ Other:

question('SQ25')

32. Do you observe any relevant cross reactions in your cysticercosis antigen tests?

- ○ No
- ○ Yes, please provide further details:

Page 09

33. Do you have positive control samples in your laboratory?

- ○ No

○ Yes, please specify:

34. How do you evaluate sensitivity and specificity of your tests?

Tick all that apply.

☐ Rely on manufacurer’s information

☐ In-house evaluation

☐ No tests performed

☐ Other:

35. Are there national ring-trials in your country to ascertain the quality of your T. solium and T. saginata tests?

○ No

○ Yes, please specify (i.e. test, organization):

36. Would you be interested in participating in an European ring trial for T. solium and T. saginata tests?

○ For T. solium tests

○ For T. saginata tests

○ For both

○ No, I would not participate

37. Do you have diagnostic tests for other Taenia spp. available?

○ No

○ Yes. Please specify:

Page 10

38. Approximately how many T. solium/saginata taniosis or cysticercosis positive samples have you had last year?

○ I don’t know

○ None

○ 1-5

○ 6-10

○ 11-50

○ 51-100

○ more than 100

39. Can you tell us exactly how many taeniosis and/or cysticercosis positive samples you had?

Preferably within the period 1990 – 2014 and using the provided excel sheets. However, every kind of reporting of cases is useful.

- ○ No
- ○ Yes

If possible, can you send us the positive cases in the file provided?

1. Click on the following link to download the excel file:

Laboratory Data Collection Sheet

2. Fill in data in the excel file
3. Save the file with the name of your laboratory as filename
4. Send the file to the following e-mail address: cystinet@gmail.com

40. Was it possible to report the positive samples?

- ○ I filled in the excel file and sent them via e-mail
- ○ I would like to provide data, but I had problems reporting it. Please contact me at this e-mail/phone: ______
- ○ No data available
- ○ Other: ______

Page 11

41. Do you have any comments?

If yes, please specify:

If you would like to send us the filled in excel sheet or any other document, article report etc. please send it to cystinet@gmail.com

42. This is the end of the questionnaire.

To submit the final version please tick the box below and click “Next”.

If you want to edit your responses later, please click on “Pause Questionnaire”.

- ○ I would like to submit all answers

Last page

Thank you for completing this questionnaire!

We would like to thank you very much for helping us.

Your answers were transmitted, you may close the browser window or tab now.

---

contact e-mail, CYSTINET – COST ACTION TD1302 - 2014
